# Echinocandins – structure, mechanism of action and use in antifungal therapy

**DOI:** 10.1080/14756366.2022.2050224

**Published:** 2022-03-16

**Authors:** Mateusz Szymański, Sandra Chmielewska, Urszula Czyżewska, Marta Malinowska, Adam Tylicki

**Affiliations:** a Department of Microbiology and Biotechnology, Laboratory of Cytobiochemistry, University of Bialystok, Bialystok, Poland; b Doctoral School of Exact and Natural Sciences, University of Bialystok, Bialystok, Poland; c Department of Organic Chemistry, Laboratory of Natural Product Chemistry, University of Bialystok, Bialystok, Poland

**Keywords:** Anidulafungin, caspofungin, micafungin, rezafungin, drug resistance, chemical modification

## Abstract

With increasing number of immunocompromised patients as well as drug resistance in fungi, the risk of fatal fungal infections in humans increases as well. The action of echinocandins is based on the inhibition of β-(1,3)-d-glucan synthesis that builds the fungal cell wall. Caspofungin, micafungin, anidulafungin and rezafungin are semi-synthetic cyclic lipopeptides. Their specific chemical structure possess a potential to obtain novel derivatives with better pharmacological properties resulting in more effective treatment, especially in infections caused by *Candida* and *Aspergillus* species. In this review we summarise information about echinocandins with closer look on their chemical structure, mechanism of action, drug resistance and usage in clinical practice. We also introduce actual trends in modification of this antifungals as well as new methods of their administration, and additional use in viral and bacterial infections.

## Introduction

Diseases caused by fungi are a serious problem. Currently, the number of people affected by fungal infections worldwide exceeds one billion^1^, and the number of deaths caused by invasive fungal species is comparable to the mortality of tuberculosis − 1.5 million each year^2^. About 90% of mortalities are caused by fungi grouped into four genera: *Candida*, *Aspergillus*, *Cryptococcus*, and *Pneumocystis*^3^ (Table 1). There are at least 17 pathogenic species complexes in the genus *Candida*, but more than 90% of infections are attributed to *Candida albicans*, *Candida glabrata*, *Candida parapsilosis*, *Candida tropicalis*, and *Candida krusei*^4^. The patients suffering from AIDS, cancer (and associated chemotherapy), leukaemia, patients on immune-compromising drug therapy, and after organ transplantation are particularly vulnerable to mycoses^5^.

**Table 1.** The estimated global annual number of selected fungal infections^1^^,^^3^.

Disease (and species)	Estimated number of infections each year globally
Fungal asthma (*Aspergillus spp.*)	10,000,000
Chronic pulmonary aspergillosis (*Aspergillus fumigatus*)	3,000,000
Fungal keratitis (*Candida spp., Aspergillus spp.*)	1,000,000
Invasive candidiasis (*Candida albicans*)	700,000
Pneumocytosis (*Pneumocystis jirovecii*)	500,000
Cryptococcal meningitis (*Cryptococcus neoformans*)	223,100
Histoplasmosis (*Histoplasma capsulatum*)	100,000
Mucormycosis (*Rhizopus oryzae*)	10,000

Until the end of the 20th century, azoles, polyenes, and flucytosine were mainly used to treat mycoses. These classes of drugs can cause serious side effects related to their hepato- and nephrotoxicity. In addition, many fungal strains have developed resistance to these antibiotics, which significantly reduces their efficacy^6^. In rare cases, cross-resistance to polyenes and azoles may occur, raising concerns about the future of antifungals targeting membrane ergosterol (polyens) and sterol synthesis (azoles)^7^. In addition, there are frequent drug-drug reactions associated with interactions of the aforementioned drugs and their metabolites in the body^8^. This has prompted the search for alternative agents to combat fungal infections. Echinocandins are a class of antifungal drugs that are fungicidal against many fungi including *Candida* species, but are fungistatic to the *Aspergillus* genus. This class of drugs has been found to cause milder side effects compared to polyenes and azoles^9^. The mechanism of action based on the inhibition of fungal-specific metabolic pathway and limited side effects have resulted in increasing interest and use of this class of drugs^10^.

The purpose of this paper is to introduce echinocandins as antifungal antibiotics for the reader, details of their chemical structure and proposed modifications, mechanism of action as well as their usage in clinical practice are also described. The use of echinocandins has improved patient outcome; however, there are reports about drug resistance of fungi to this class of antibiotics. Therefore, an additional aim of this study is to summarise the latest information on the phenomenon of fungal drug resistance to echinocandins.

## History of the discovery of echinocandins

In 1974, in Switzerland the first antifungal drug of the echinocandin class – echinocandin B was discovered, which showed good antifungal properties but at the same time strong haemolytic effects. To counteract this, cilofungin, a semi-synthetic analogue of echinocandin B with a 4-octyloxybenzoate side chain, was synthesised. This compound significantly reduced haemolytic activity while retaining antifungal properties^11^. However, cilofungin was withdrawn from Phase II clinical trials due to the poor water solubility and toxicity of its co-solvent^12^. An important step in echinocandin research was the discovery of pneumocandin A_0_ and pneumocandin B_0_, of which pneumocandin B_0_ was used to synthesise a new antifungal agent^13^ (Table 2).

**Table 2.** Names and physical properties of selected echinocandins^12^^,^^14–16^.

Echinocandins	Synonyms (MeSH Entry terms) or IUPAC Name	Summary formula	Molecular weight (g/mol)	Log P	Solubility in water (mg/ml)
Caspofungin	(1-[(4R,5S)-5-[(2-aminoethyl)amino]15-N2-(10,12-dimethyl-1-oxotetradecyl)-4-hydroxy-L-ornithine]-5-[(3R)-3-hydroxy-L-ornithine]-pneumocandin B_0_ diacetate	C_52_H_88_N_10_O_15 _* 2 C_2_H_4_O_2_	1093.30	**-**3.88	28
Micafungin	(1-[(4R,5R)-4,5-dihydroxy-N2-[4-[5-[4-(pentyloxy)phenyl]-3-isoxazolyl]benzoyl]-L-ornithine]-4-[(4S)-4-hydroxy-4- [4-hydroxy-3- (sulfooxy)phenyl]- L-threonine]pneumocandin A0 sodium salt	C_56_H_70_N_9_NaO_23_S	1292.26	**-**1.50	>200
Anidulafungin	(1-[(4R,5R)-4,5-dihydroxy-N2-[[4"-(pentyloxy)[1,1′:4′,1"-terphenyl]-4-yl]carbonyl]L-ornithine]echinocandin B	C_58_H_73_N_7_O_17_	1140.30	2.90	0.05
Rezafungin	2-[[(3S,6S,9S,11R,15S,18S,20R,24S,25S,26S)-6-[(1S,2S)-1,2-dihydroxy-2-(4-hydroxyphenyl)ethyl]-11,20,25-trihydroxy-3,15-bis[(1R)-1-hydroxyethyl]-26-methyl-2,5,8,14,17,23-hexaoxo-18-[[4-[4-(4-pentoxyphenyl)phenyl]benzoyl]amino]-1,4,7,13,16,22-hexazatricyclo[22.3.0.09,13]heptacosan-21-yl]oxy]ethyl-trimethylazanium	C_63_H_85_N_8_O_17_^+^	1226.40	2.90	>150

Log P – partition coefficient.

In 1992, caspofungin acetate was first synthesised from pneumocandin B_0_ and approved for clinical trials^13^. The U.S. Food and Drug Administration (FDA) approved caspofungin in January 2001 as a drug for the prevention of fungal infections in adult patients, and the first medical preparations containing caspofungin were introduced to the U.S. market the same year. In Europe, the antibiotic was approved for therapy in 2002 under the trade name CANCIDAS (manufacturer: Merck & Co. Inc., USA, MK-0991)^17^. In July 2008, it was approved for the treatment of children over 3 months of age^18^.

The precursor of micafungin, which was FR901379, was discovered in Japan by Fujisawa Pharmaceutical. To stop FR901379-induced reticulocyte lysis, it underwent various modifications. This resulted in the compound FK463 called micafungin, which had reduced haemolytic activity and was potent against *Candida* and *Aspergillus* species^19^. The drug was approved in the U.S. in 2005, and in Europe in 2008. The compound was also approved for the treatment of invasive candidiasis in patients younger than 4 months of age^20^. The first formulations of micafungin were produced in Japan and sold under the trade name MYCAMINE (manufacturer: Astellas Pharma Inc., Japan, FK-463)^17^.

Another of the echinocandins, anidulafungin was obtained by optimising the chemical structure of echinocandin B. Replacement of the naturally occurring fatty acid side chain with an alkoxytriphenyl side chain reduced the haemolytic effect of echinocandin B^17^. Anidulafungin, as an antifungal compound for the treatment of oesophageal candidiasis, candidemia and deep tissue candidiasis, was approved in the US in 2006 and marketed under the trade name ERAXIS. In Europe, the drug was marketed a year later under the trade name ECALTA (manufacturer: Pfizer Inc. UK)^21^.

The "ReSTORE" study (ClinicalTrials.gov registration number NCT03667690)^16^ of rezafungin (CD101), the newest member of the echinocandin class, is currently ongoing. Two post-study Phase III clinical trials are underway to evaluate the efficacy of rezafungin for the treatment of candidemia and invasive candidiasis and for the prevention of invasive fungal infections caused by *Candida*, *Aspergillus* and *Pneumocystis* species (patient recruitment for the trials began in October 2018, with planned completion of the trial set for August 2021)^22^. Rezafungin is a structural analogue of anidulafungin in which the hemiaminal region at the C5 ornithine position has been replaced with a choline amine ether. With this modification, the stability of the drug in solutions and pharmacokinetics are improved, as well as the drug's action in the body is prolonged^10^. It is also possible to treat infections caused by fungal strains resistant to other antibiotics such as azoles with rezafungin^23^.

## Chemical structure of selected echinocandins

**Figure 1.**
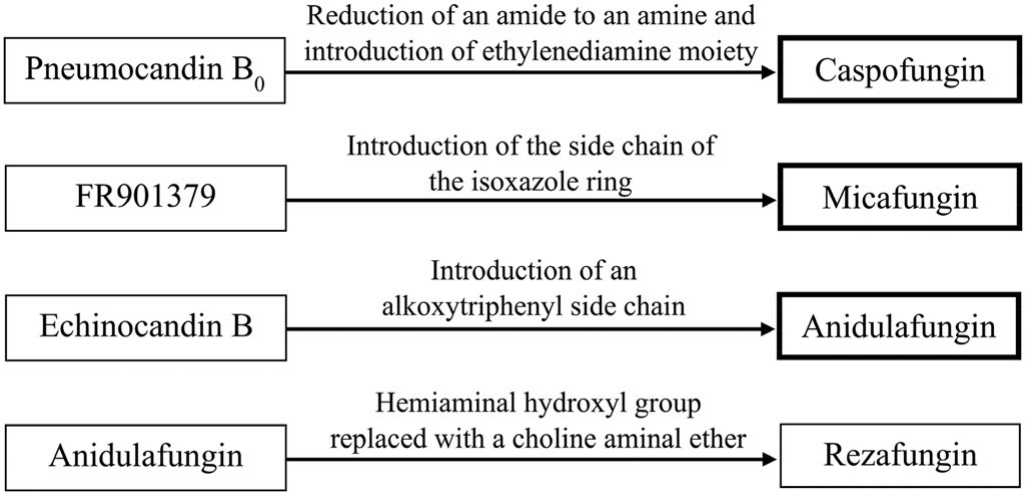
Echinocandin precursors and their structural modifications resulting in the three representatives approved for clinical use (bold boxes), rezafungin is under phase III clinical trials.

**Figure 2.**
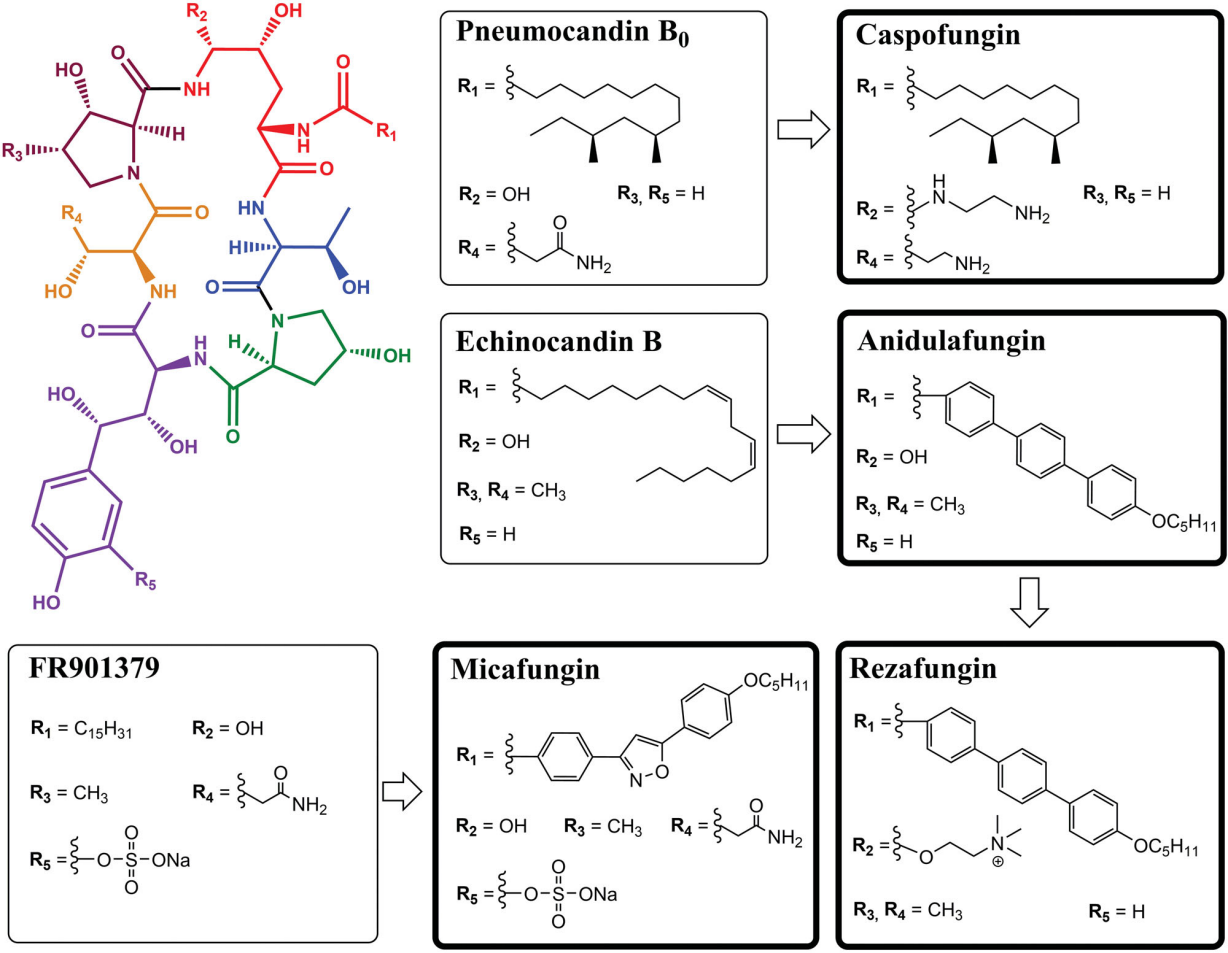
Structure of selected compounds of the echinocandin class. Thin boxes indicate the natural product precursors derived from fungal metabolism, bold boxes distinguish semi-synthetic antibiotics.

Echinocandins such as pneumocandin A_0_ and B_0_, echinocandin B, and FR901379 are naturally synthesised by filamentous fungi. Caspofungin, micafungin, anidulafungin and rezafungin are semi-synthetic cyclic lipopeptides with antifungal activity (Figure 1). They have a core composed of a cyclic hexapeptide and are acylated with a different fatty acid attached to the α-amino group of dihydroxyornithine (Figure 2). This lipid residue is required to anchor the drug to the cell membrane and is therefore essential for bioactivity^11^.

### Echinocandin B, pneumocandin B_0_ and synthesis of caspofungin

Echinocandin B (Figure 2) is a major lipopeptide antifungal antibiotic from a complex produced by *Aspergillus nidulans* and *Aspergillus rugulosus*. It contains a linoleic acid side chain and a hexacyclic peptide core built by various amino acid residues such as 3,4-dihydroxyhomotyrosine, 3-hydroxy-4-methylproline, 4,5-dihydroxyornithine, 4-hydroxyproline and two threonine residues^24^. These amino acid residues have a significant effect on antifungal activity and determine the physicochemical properties of the echinocandin B nucleus. For example, modified proline and homotyrosine residues are essential for the antifungal efficacy of echinocandins^25^. Hydroxyl groups at the three amino acid residues forming the cyclic lipopeptide core, improve the solubility of the drug in water and aid its stability in solutions^12^. Taking this into account, during the synthesis of newer echinocandin B derivatives, the core was kept unchanged or underwent minor modifications^26^ (Figure 1). The hydrophobic fatty acid chain attached to the echinocandin B core is crucial for antifungal activity because it acts as a "hook" that allows the drug to anchor in the fungal cell membrane^12^.

**Figure 3.**
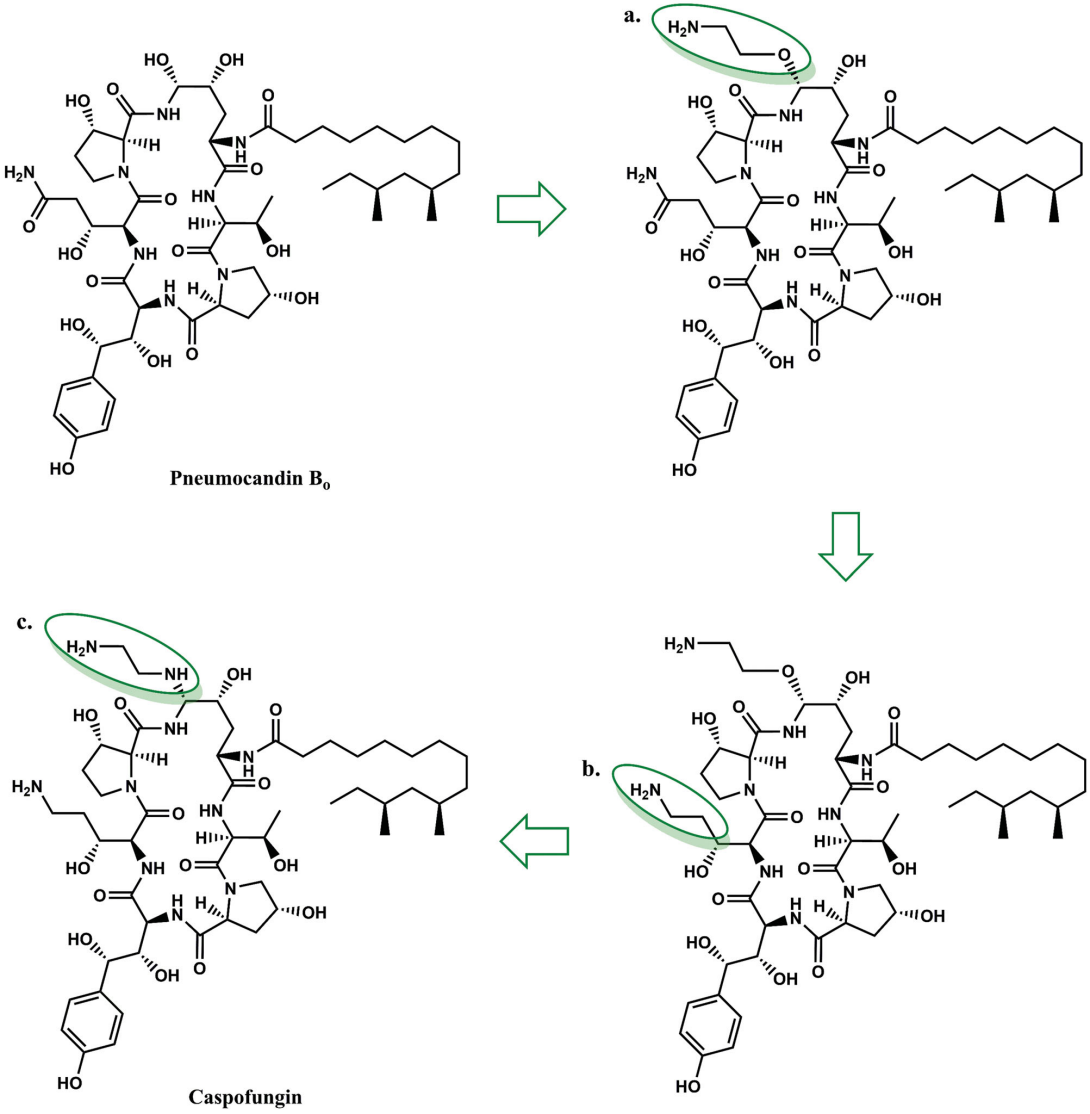
Medicinal chemistry progression from pneumocandin B_0_ to caspofungin. The differences in the structure of pneumocandin B_0_ that lead to caspofungin are marked in green circles as: (a) aminoethyl ether, (b) 3-hydroxyglutamine reduction site, (c) ethylenediamine (according to^13^^,^^27^).

**Figure 4.**
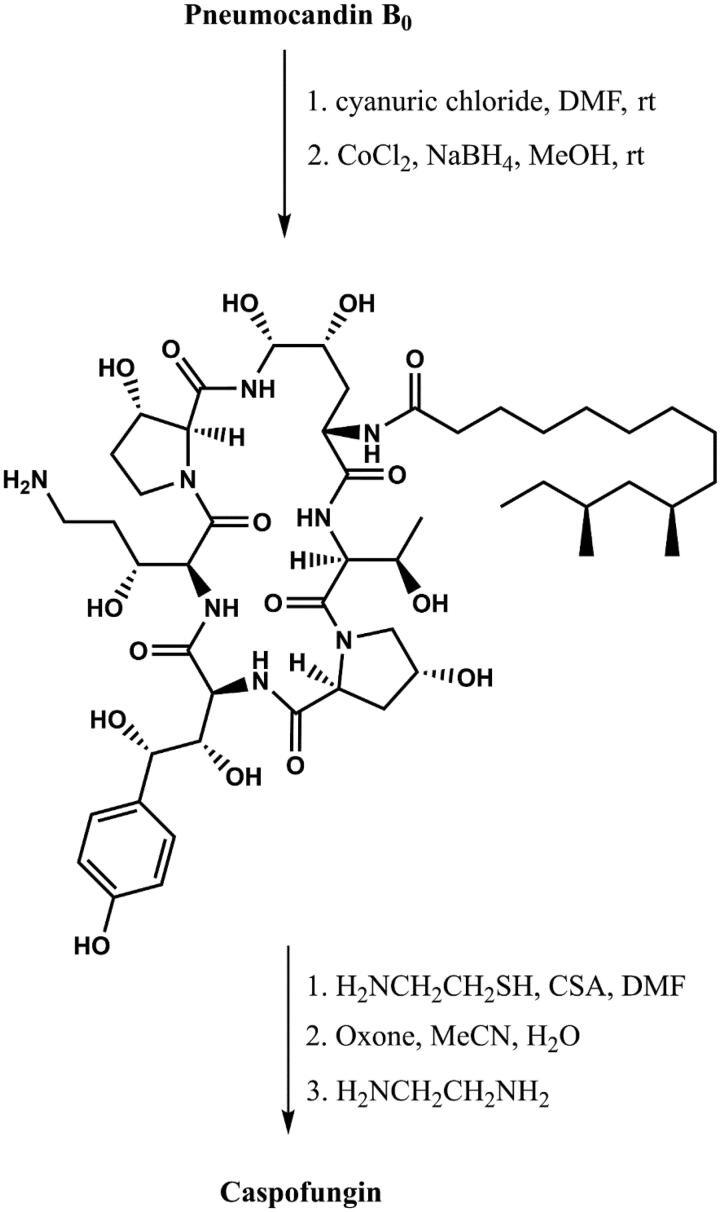
Synthesis of caspofungin (according to^29^).

**Figure 5.**
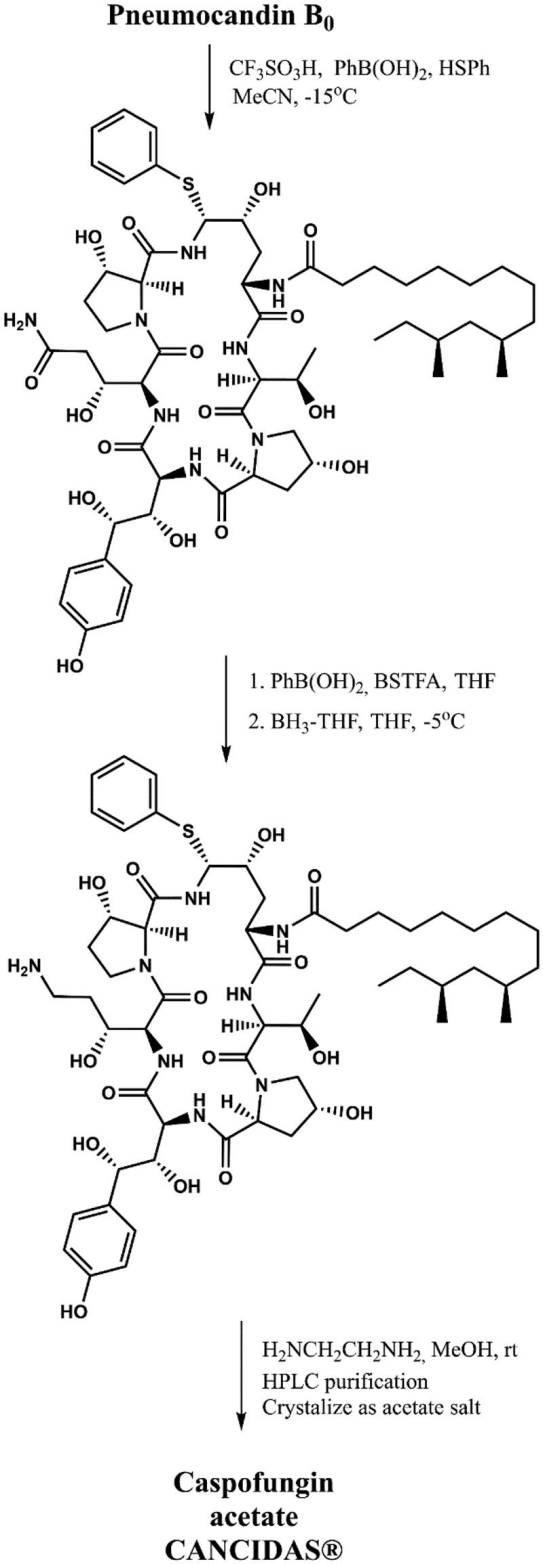
Improved synthesis of caspofungin acetate - CANCIDAS® (according to^29^).

**Figure 6.**
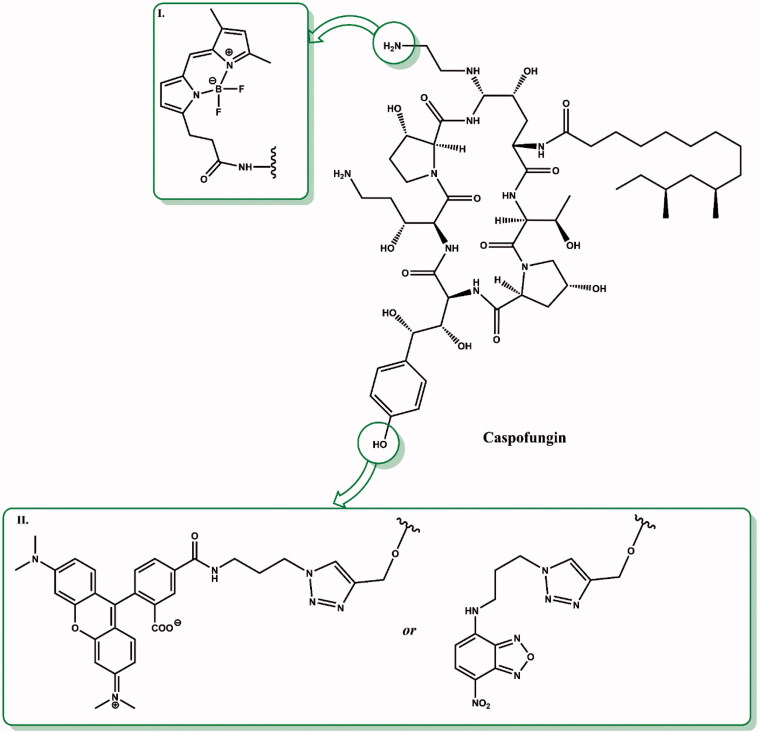
Fluorescent derivatives of caspofungin. Structural elements changed in the structure of caspofungin are shown in green boxes (according to^14^^,^^32^).

**Figure 7.**
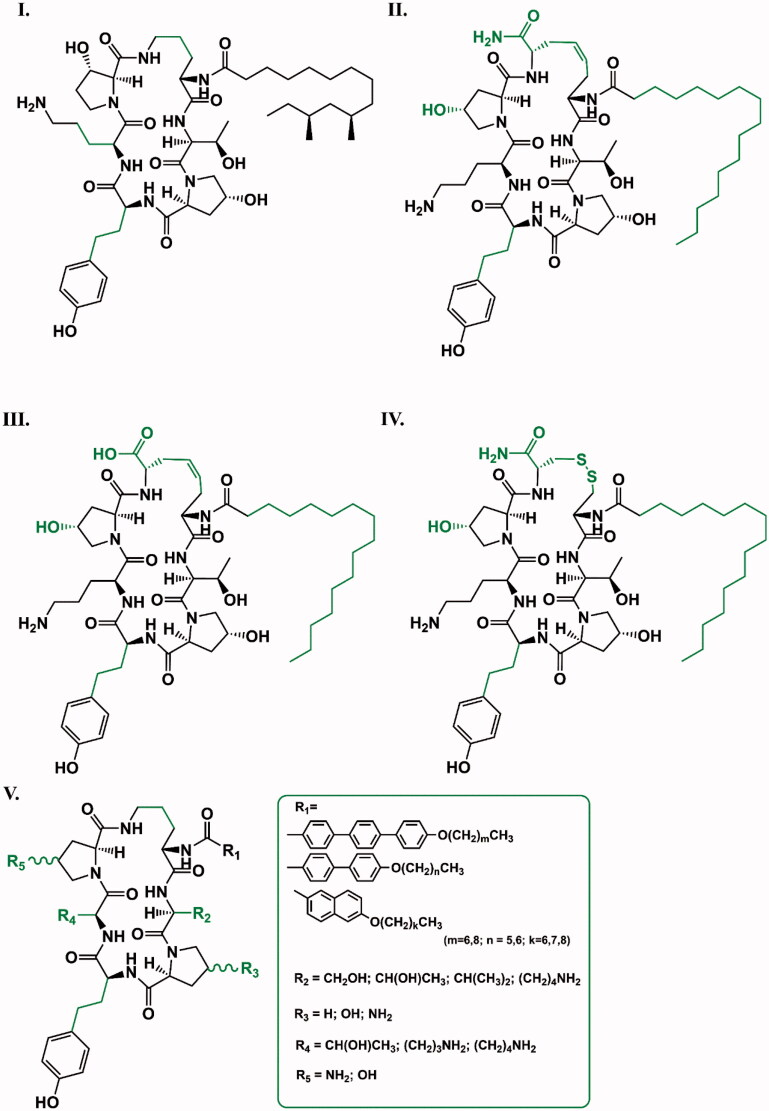
Completely synthetic caspofungin derivatives. The differences in the structure of new compounds relative to caspofungin are shown in green (II., III., IV. according to^25^; I. according to^32^; V. according to^33^).

Caspofungin is a cyclic, semisynthetic water-soluble lipopeptide (Figure 3). This compound is a derivative of the naturally occurring hexapeptide in *Glarea lozoyensis*, pneumocandin B_0_^26^^,^^28^. This compound was developed as a result of a four-year program of medicinal chemistry to obtained a derivative with improved water solubility that would facilitate the development of an intravenous formulation. Initial attempts to optimise the structure produced derivatives with a cationic aminoethyl ether group, as well as a 3-hydroxyornithine in place of the 3-hydroxyglutamine moiety (Figure 3). These modifications increased water solubility, stability as well as activity against *Candida* and *Aspergillus* spp.^27^. Initial studies had suggested that improved activity was caused by the presence of cationic groups that can form ion pairs with the negatively charged phosphate group of the phospholipid. The consequence was an increase in the concentration of lipopeptide in the cell membrane where the target, glucan synthase, is located. Replacement of the aminoethyl ether with ethylene diamine resulted in the identification of caspofungin, which was synthesised via a two-step modification of the peptide core of pneumocandin B_0_. First carboxamide of 3-hydroxyglutamine was selectively reduced to an amine by a two-step method. Second, condensation of the hemiaminal group with ethylenediamine furnished caspofungin^29^ (Figure 4). After improving the methodology, caspofungin acetate – CANCIDAS® was obtained by a very efficient three-step synthesis^29^ (Figure 5). Despite the success of the medicinal chemistry program, new analogues of caspofungin are still under development. To study echinocandins cellular biology in *Candida* species, some fluorescent caspofungin’s derivatives were developed^30^^,^^31^ (Figure 6). It should be emphasised that some of new caspofungin derivatives do not show biological activity (such as, for example, derivatives obtained by cross-metathesis with a larger peptide ring size than in naturally occurring echinocandins)^32^ (Figure 7). The possibility of designing macrocyclic compounds by total synthesis is very promising. The use of mild conditions in efficient reactions is an interesting alternative to the semi-synthetic method. The application of total synthesis enables to conveniently plan a wide variety of compounds and to study the relationship between structure and biological activity^34^.

### Synthesis of micafungin

**Figure 8.**
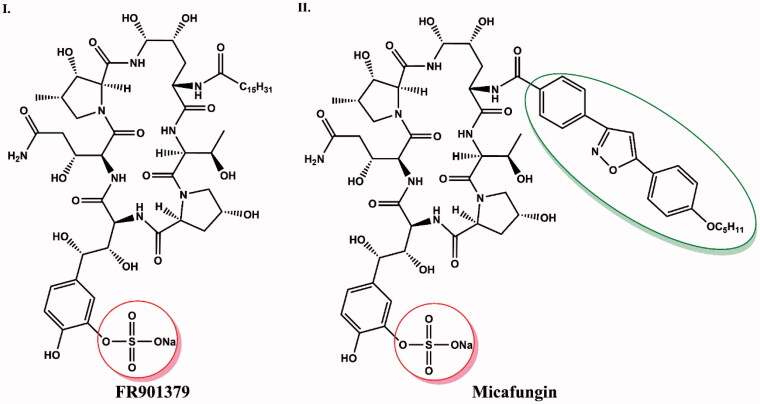
Structure comparison of FR901379 (I) and micafungin (II). The side chain of the isoxazole ring that distinguishes micafungin from FR901379 is shown in green circle. The sulphate group responsible for increasing water solubility of both compounds is highlighted in red.

Another group of echinocandins are compounds with a sulphate moiety instead of a hydroxyl group in dihydroxyhomotyrosine. Micafungin (Figure 8) was formed by enzymatic deacylation of a naturally occurring hexapeptide derived from *Coleophoma empetri* (FR901379), to which an optimised *N*-acylated side chain was then introduced^35^. The side chain contains an isoxazole ring substituted by 3,5-diphenyl^36^. This modification reduces the haemolytic activity of the drug compared to FR901379 while retaining the antifungal properties of the precursor^19^. The sulphate group in the dihydroxyhomotyrosine side chain in the compound structure increase the water solubility of micafungin^37^.

### Modification of echinocandin B – anidulafungin and rezafungin

**Figure 9.**
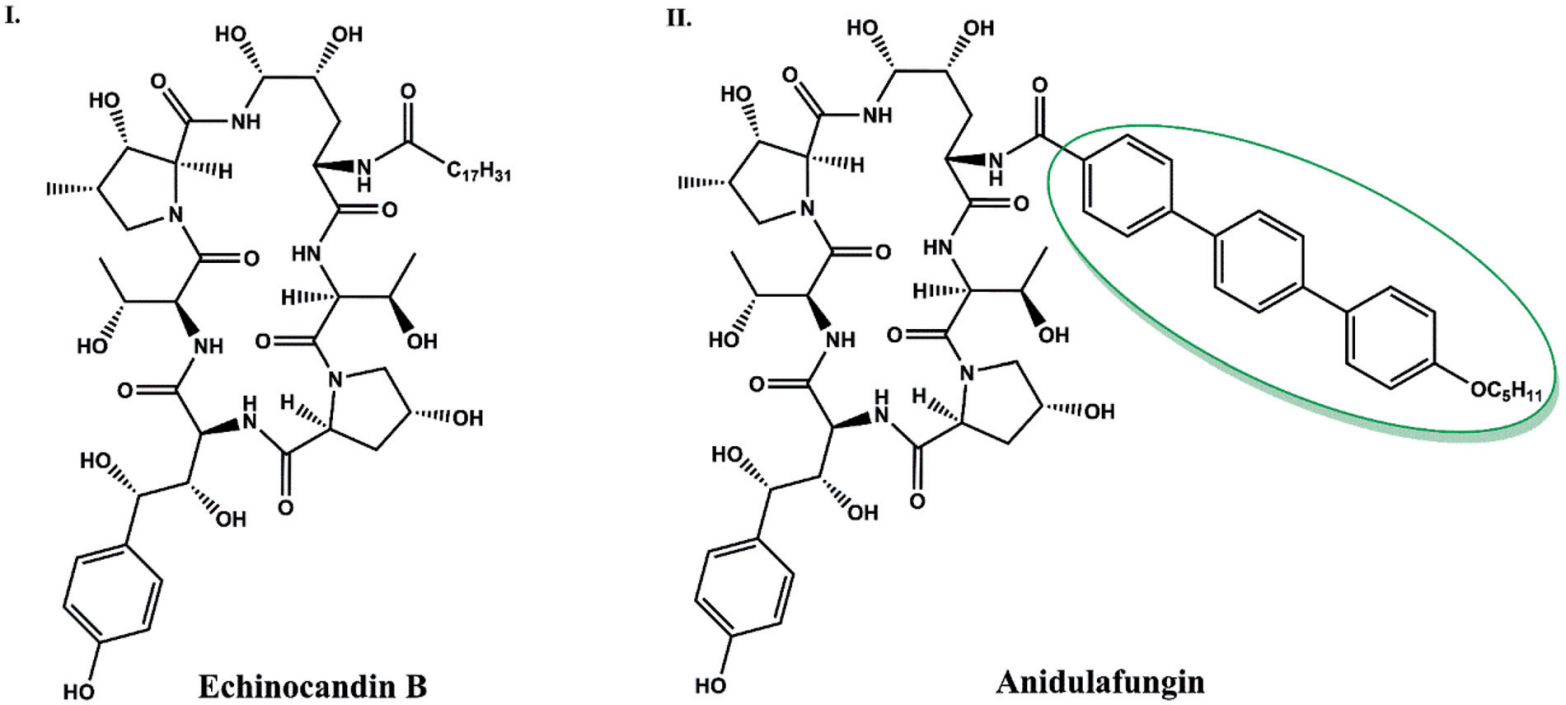
Structure comparison of echinocandin B (I) and anidulafungin (II). The alkoxytriphenyl side chain that distinguishes the structure of anidulafungin from echinocandin B is shown in green circle.

**Figure 10.**
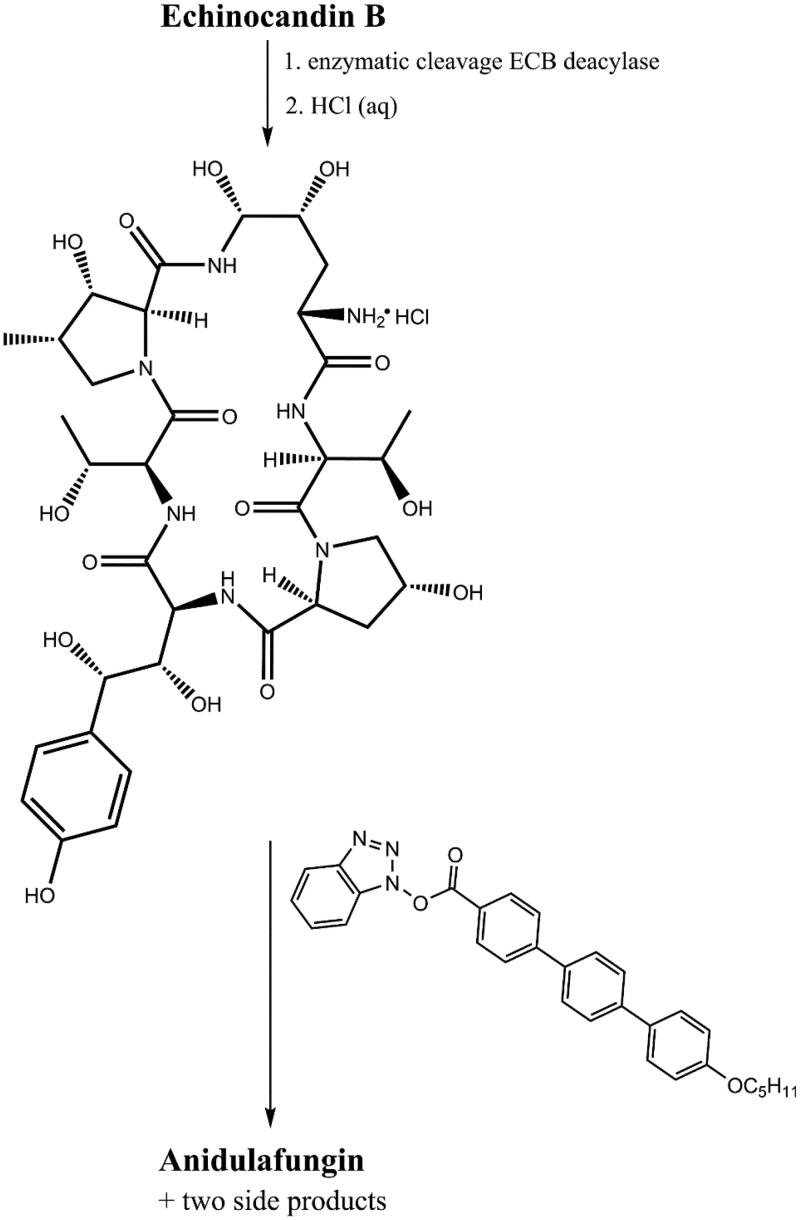
Synthesis of anidulafungin (according to^39^).

**Figure 11.**
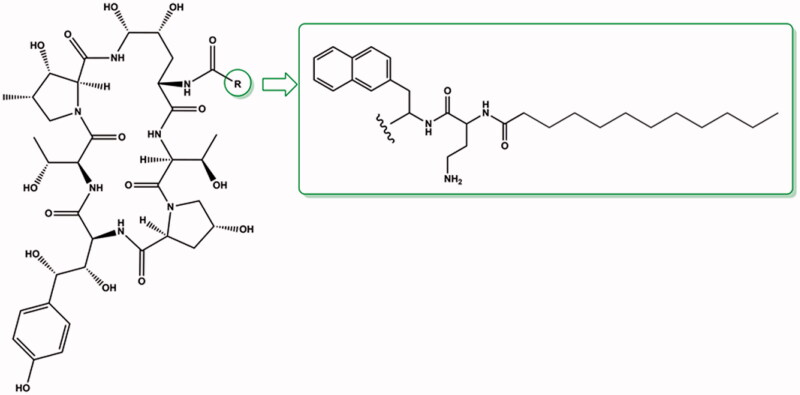
Structure of new semi-synthetic derivative of anidulafungin (according to^42^).

Anidulafungin is a semi-synthetic derivative of echinocandin B^9^ (Figure 9). Anidulafungin, as well as other echinocandins, consists of a peptide nucleus of echinocandin B, composed of amino acid residues. Enzymatical deacylation of echinocandin B using *Actinoplanes utahensis* culture introduced hydrochloride salt in place of the linoleoyl side chain. Next, this HCl salt was reacted with the activated ester to form anidulafungin^38^ (Figure 10). The introduction of an alkoxytriphenyl side chain in place of the alkyl chain of echinocandin B reduces the haemolytic properties of the drug and has a key effect on the intercalation of anidulafungin with the fungal cell membrane, but reduces the solubility of the drug in water^12^^,^^38^. Poor solubility and low oral bioavailability of anidulafungin cause a necessity of administration by intravenous injection^40^^,^^41^. To increase the solubility, derivatives with a modified side chain have recently been prepared, and one of them is particularly promising^42^ (Figure 11). According to primary tests, it shows high activity against *C. albicans* and *C. krusei* with better water solubility and lower toxicity *in vitro* in murine macrophages (RAW264.7) than anidulafungin^41^. The observed effect may be particularly important considering the therapy of immunocompromised patients.

**Figure 12.**
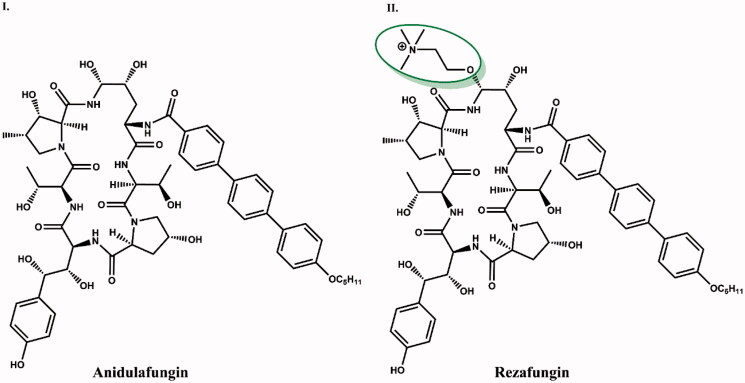
Structure comparison of anidulafungin (I) and rezafungin (II). Choline amine ether at the C5 ornithine position distinguishing the two compounds is shown in green circle.

Anidulafungin is the precursor of rezafungin (CD101) with an additional choline ether^15^ (Figure 12). The choline amine ether moiety at the C5 position of ornithine increased the stability of the compound (reduced degradation of the compound in the hemiaminal region), half-life, solubility^10^^,^^22^^,^^23^. During degradation processes, anidulafungin may undergo a ring-opening process in which the hemiaminal cleaves to form a linear peptide having a terminal amide and aldehyde from the hydroxyl C5 ornithine residue forming reactive intermediates that undergo further metabolism in the body and persist until eliminated in the faeces. The increased chemical stability of rezafungin prevents ring opening resulting in the absence of reactive metabolites that may contribute to toxicity^15^^,^^43^.

## Mechanism of echinocandin action

Fungal cell wall components include β-(1,3)-d-glucans, β-(1,4)-d-glucans, β-(1,6)-d-glucans, α-glucans, chitin, mannan, and a variety of glycoproteins^42^. Glucans are particularly important components in maintaining the cell wall integrity of *Candida spp*. and *Saccharomyces spp.* accounting for approximately 50–60% of the cell wall components of these fungi. Besides being an important component of fungal cell wall structure, β-(1,3)-d-glucan is not found in animal cells, so its synthesis is a good target for antifungal antibiotics^44^.

**Figure 13.**
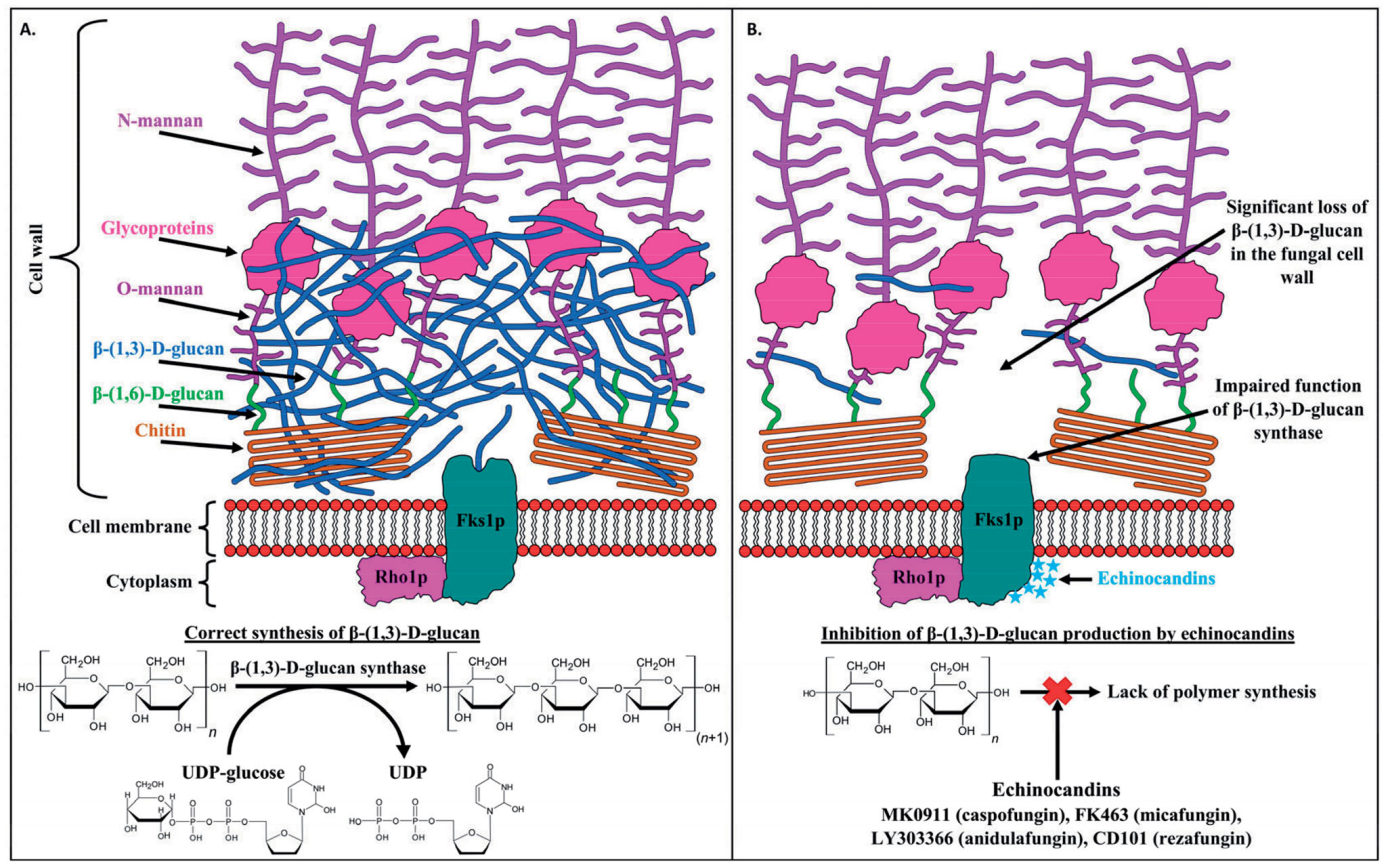
Mechanism of action of echinocandins. (A) normal production of β-(1,3)-d-glucan, (B) echinocandins acting on the FKS1p subunit non-competitively inhibit glucan synthase activity disrupting β-(1,3)-d-glucan synthesis, leading to fungal cell death caused by cell wall instability.

The synthesis of β-(1,3)-d-glucan is catalysed by UDP-glucose (1,3)-d-glucan-β-(3)-d-glucosyltransferase, referred to as β-(1,3)-d-glucan synthase (EC 2.4.1.34)^45^. This enzyme uses UDP-glucose as a reaction substrate to form β-(1,3)-d-glycosidic bonds^46^. The enzyme is a transmembrane heteromeric glycosyltransferase consisting of at least two subunits. The Fks1p subunit (encoded by the *FKS1*, *FKS2*, and *FKS3* genes) has a catalytic function, while the Rho1p subunit (belonging to the GTPase family) has a regulatory function. Echinocandins binding non-competitively to the Fks1p subunit of the enzyme inhibits its activity^47^^,^^48^. Blocking β-(1,3)-d-glucan biosynthesis leads to structural abnormalities of the fungal cell wall (Figure 13), resulting in growth inhibition or death by imbalance in osmotic pressure^49^. The fungicidal or fungistatic effects of echinocandins have been confirmed for most species of the *Candida* and *Aspergillus* genera^50^.

## Antifungal activity, metabolism and medicinal use of echinocandins

The range of MIC values of echinocandins varies from 0.007 μg/ml to 32 μg/ml depending on the *Candida* species. Overall, anidulafungin shows the most potency against most *Candida* pathogens^51^ (Table 3).

**Table 3.** Range of MIC and MEC values (μg/ml) of echinocandins against selected *Candida* and *Aspergillus* species^52–55^.

Species	Caspofungin	Micafungin	Anidulafungin
*Candida* species	Range of MIC values		
*C. albicans*	0.07–0.5	0.008–4	0.008–2
*C. glabrata*	0.015–8	0.008–32	0.008–4
*C. parapsilosis*	0.015–4	0.015–4	0.015–8
*C. tropicalis*	0.007–8	0.008–8	0.015–2
*C. krusei*	0.015–1	0.015–1	0.030–2
*C. guillermondii*	0.030–8	0.015–8	0.030–4
*C. lusitianiae*	0.030–1	0.015–8	0.008–1
*Aspergillus species*	Range of MEC values		
*A. fumigatus*	0.015–1	0.008–0.06	0.008–0.125
*A. flavus*	0.008–0.03	0.008–0.003	0.008–0.015
*A. terrus*	0.015–1	0.008–0.03	0.008–0.0125

MIC: minimum inhibitory concentration; MEC: minimum effective concentration.

Echinocandins are recommended as a treatment for patients suffering from an invasive infection caused mainly by *Candida*, *Aspergillus* species and some other pathogenic fungi^50^ (Table 4). These antibiotics also act on biofilm-forming yeasts especially on the *Candida* genus^64^. Relative to *Candida* species, echinocandins exhibit fungicidal activity manifested by significant cell enlargement and distortion, which contributes to inhibition of cell proliferation. Against *Aspergillus* species (*A. fumigatus*, *A. flavus*, *A. niger*, and *A. terreus*), echinocandins exert fungistatic effects by causing irregular growth of the hyphae with multiple branched tips and distended cells, preventing the pathogen from spreading beyond the initial site of infection^65–67^. Echinocandins are also active against some species of *Penicillium* and *Paecilomyces*. To a lesser extent, they show activity against *Madurella*, *Wangiella*, *Sporothrix*, *Exophiala*, *Scedosporium*, *Pseudallescheria* and *Fonsecaea* genera^68^. These antibiotics used without additional antifungal compounds are not effective for the treatment of mycoses caused by *Mucorales*, *Cryptococcus*, *Fusarium*, *Rhizpous* and *Trichosporon* genera^69^^,^^70^. The cell wall of the above-mentioned fungal genera contains mainly β-(1,6)-d-glucans, which limits their sensitivity to echinocandins^69^. The activity of these antibiotics against representatives of the genera *Histoplasma*, *Blastocystis* and *Coccidioides* is also limited^71^.

**Table 4.** Susceptibility of selected fungal species to different antifungal drugs^38^^,^^56–63^.

	Polyenes	Azoles		Echinocandins		
Fungi	AmB	FLU	VOR	CAS	MIC	AND
*Candida albicans*	**+**	**+**	**+**	**+**	**+**	**+**
*Candida glabrata*	**+**	**+/−**	**+**	**+**	**+**	**+**
*Candida parapsilosis*	**+**	**+**	**+**	**+**	**+**	**+**
*Candida tropicalis*	**+**	**+**	**+**	**+**	**+**	**+**
*Candida krusei*	**+**	**−**	**+**	**+**	**+**	**+**
*Candida lusitaniae*	**−**	**+**	**+**	**+**	**+**	**+**
*Aspergillus fumigatus*	**+**	**−**	**+**	**+**	**+**	**+**
*Aspergillus flavus*	**+/−**	**−**	**+**	**+**	**+**	**+**
*Aspergillus niger*	**+**	**−**	**+**	**+**	**+**	**+**
*Aspergillus terreus*	**−**	**−**	**+**	**+**	**+**	**+**
*Acremonium spp.*	**+**	**−**	**+**	**−**	**−**	**−**
*Alternaria spp.*	**+**	**−**	**+**	**−**	**−**	**−**
*Blastomyces spp.*	**+**	**+**	**+**	**+/−**	**+/−**	**+/−**
*Coccidioides spp.*	**+**	**+**	**+**	**+/−**	**+/−**	**+/−**
*Cryptococcus neoformans*	**+**	**+**	**+**	**−**	**−**	**−**
*Curvularia spp.*	**+**	**+**	**+**	**+**	**+**	**+**
*Fusarium spp.*	**+/−**	**−**	**+**	**−**	**−**	**−**
*Histoplasma spp.*	**+**	**+**	**+**	**+/−**	**+/−**	**+/−**
*Mucorales*	**+**	**−**	**+**	**−**	**−**	**−**
*Rhizpous spp.*	**+**	**+/−**	**+**	**−**	**−**	**−**
*Scedosporium spp.*	**+/−**	**−**	**+/−**	**−**	**−**	**−**
*Trichoderma spp.*	**+**	**−**	**+**	**+**	**+**	**+**
*Trichosporon spp.*	**+**	**−**	**+**	**−**	**−**	**−**
*Zygomycetes*	**+/−**	**−**	**−**	**−**	**−**	**−**

AmB: amphotericin B; FLU: fluconazole; VOR: voriconazole; CAS: caspofungin; MIC: micafungin; AND: anidulafungin.

"+" fungi susceptible to a specific antibiotic; "−" resistant species; "**+/−**" organisms showing variable response indicated drugs.

Echinocandins are given to patients intravenously (over 1 h infusion), because they are poorly absorbed in the gastrointestinal tract (less than 3% of the antibiotic is absorbed after oral administration). They also cannot penetrate the central nervous system or the eyes, and for this reason are not used to treat intraocular inflammation or fungal meningitis^72^. These antibiotics bind strongly to proteins (97–99%), leading to lower concentrations of the drug available in serum and tissues^36^^,^^59^. The half-life ranges from 9 to 133 h (Table 5), so they are administered once daily (for FDA approved ones) or once weekly (rezafungin). The clinical response of the patient affects the length of treatment. Most patients should be treated 14 days or no more than 7 days after the resolution of symptoms^5^. Echinocandins are metabolised mostly in the liver as well as in the adrenal glands and spleen through hydrolysis and *N*-acetylation. Their metabolites are excreted mainly in the bile and faeces^18^^,^^36^. Echinocandins are found in the highest concentrations in the liver, spleen, intestine, kidney, and lung. In these tissues, their concentrations can be two to sixteen times higher than in plasma^79^. Echinocandins do not affect the P-glycoprotein family and are weak substrates for cytochrome P450^72^.

**Table 5.** Pharmacokinetics of echinocandins for adult patients^18^^,^^48^^,^^54^^,^^60^^,^^73–78^.

Echinocandin	C_max_, mg/L	AUC_0–24_, mg h/l	t½, h	CL_t_, l/h	V_d_, L	Binding to proteins, %	Excretion
Caspofungin (70 mg LD/ 50 mg DD)	12.09	97.63	9–11	0.63	9.67	97	35% in faeces, 41% in urine
Micafungin (100 mg DD)	7.20	132.60	11–17	1.30	25.60	99	40% in faeces, 15% in urine
Anidulafungn (200 mg LD/100 mg DD)	7.20	110.30	24–26	0.96	35.20	99	30% with faeces, 1% with urine
Rezafungin (400 mg LD/200 mg DD)	22.70	1160	129–133	0.23	35.90	99	38% in faeces, 14% in urine

AUC_0-24_: area under plasma concentration-time curve; CL_t_: total clearance; C_max_: maximum concentration; t½: half-life; V_d_: volume of distribution; LD: loading dose; DD: daily dose.

The administered dose in geriatric patients does not require appropriate adjustment. The elderly do not exhibit changes in pharmacokinetics in comparison to younger patients (dose adjustment is not required)^80^. Echinocandins are not removed renally, so they can be safely administered to elderly patients with renal impairment and patients undergoing dialysis. If a patient is taking several pharmaceuticals during echinocandins therapy, it is not necessary to adjust these medications because echinocandins have little drug-drug interaction. The exception is the special dosage adjustment of caspofungin in patients with hepatic impairment^80^^,^^81^.

### Caspofungin

Caspofungin besides many *Candida* and *Aspergillus* species, also has activity against *Pneumocystis jirovecii*. Its activity against *C. parapsilosis* and *C. guilliermondii*, as well as *Trichosporon beigelii*, *Rhizopus arrhizus* and *Fusarium spp.* is moderate^17^. Studies have also shown antibacterial activity of caspofungin against *Enterococcus faecium*^82^. Caspofungin has nonlinear pharmacokinetics, has an affinity for plasma proteins (97%) and has a half-life of 9–11 h in the human body (Table 5), allowing for once-daily dosing. The drug is metabolised in the liver by hydrolysis and *N*-acylation, and the metabolites are excreted mainly in the faeces (35%) and urine (41%)^72^^,^^83^. Some caspofungin undergoes spontaneous chemical degradation caused by peptide ring-opening^84^. A small amount of caspofungin is excreted unchanged in the urine (2%)^83^. In clinical practice, caspofungin is used in patients with invasive aspergillosis (caused by *A. fumigatus*) and associated neutropenia, as well as in cancer patients, HIV-infected patients, allogeneic haematopoietic stem cell transplant patients, and organ transplant patients, for preventing of fungal infections^85^^,^^86^. It may also be used to treat pleural infections, candidemia, oesophageal candidiasis, peritonitis, intra-abdominal abscesses, and abdominal infections caused by *Candida* species^13^. Caspofungin is used for treatment against voriconazole- and polyene-resistant *Aspergillus fumigatus*^9^^,^^49^. The recommended dosing regimen in adults consists of a single saturating dose of 70 mg on the first day, followed by 50 mg daily, administered over 1 h, reaching an initial therapeutic plasma concentration of 1 μg/ml^87^^,^^88^ (Table 6). Paediatric patients receive a saturating dose of 70 mg and a maintenance dose of 50 mg^14^^,^^72^. Caspofungin may reduce concentrations of rifampicin and tacrolimus in human serum^88^. In the absence of treatment response or concomitant use of cytochrome P450 inducing drugs (e.g. rifampin, efavirenz, dexamethasone), the daily dose of caspofungin should be increased to 70 mg^18^. In patients with hypoalbuminemia and liver failure, caspofungin doses should be individually adjusted^91^. Inhalation administration of the drug is also being studied, which according to data from experiments shows better antifungal properties. Additionally, the inhaled form can be used once a week and has lower hepatotoxicity than intravenous form administered once at high concentrations^92^. It is possible to treat animals such as cats suffering from invasive fungal mucositis and sinusitis caused by *Aspergillus fumigatus* with caspofungin^93^.

**Table 6.** Characteristics of echinocandins as medical preparations^5^^,^^10^^,^^17^^,^^18^^,^^22^^,^^51^^,^^89^^,^^90^.

Active pharmaceutical ingredient	Physical form	Support substances	Reconstituting using	Storage	Dosage	Treated diseases
Caspofungin acetate	Lyophilised white powder, freely soluble in water (reconstitution required)	Sucrose, mannitol, acetic acid, NaOH	0.9% sodium chloride solution or sterile water	At temperatures less than or equal to 25 °C for 24 hours or at 2 to 8 °C for 48 hours	70 mg (saturation dose), then 50 mg daily (maintenance dose); 50 mg daily*	Fungal infections in patients with fever and neutropenia, candidemia, invasive aspergillosis (in patients resistant or intolerant to other therapies); oesophageal candidiasis, intra-abdominal abscesses, peritonitis, and pleural space infections
Micafungin sodium	Lyophilised powder, freely soluble in water (reconstitution required)	Lactose, citric acid, NaOH	0.9% sodium chloride solution or 5% dextrose	Stable for 24 hours at room temperature when protected from light	No saturating dose, 100 or 50 mg daily; No saturating dose, 150 mg daily*	Candidemia; oesophageal candidiasis, intra-abdominal abscesses, peritonitis, and pleural space infections
Anidulafungin	Lyophilised powder, insoluble in water (reconstitution required)	Fructose, mannitol, polysorbate 80, tartaric acid, NaOH or HCl	0.9% sodium chloride solution, 5% glucose solution, 20% dehydrated alcohol in water	In the refrigerator up to 24 hours	200 mg (saturating dose), then 100 mg daily (maintenance doses); 100 mg (saturation dose), then 50 mg daily (maintenance dose)*	Candidemia; oesophageal candidiasis, intra-abdominal abscesses, peritonitis, and pleural space infections
Rezafungin acetate	Lyophilised powder, freely soluble in water (reconstitution required)	Mannitol, polysorbate 80, histidine	0.9% sodium chloride solution or sterile water	Stable for over an year and shows minimal degradation	400 mg first week followed by 200 mg once weekly	Invasive candidiasis, aspergillosis and pneumocystis pneumonia

*Underlined special dosages are applied for oesophageal candidiasis.

### Micafungin

Micafungin has potent antifungal activity against a broad spectrum of *Candida* species and azole-resistant *Aspergillus* species^19^. It binds significantly (>99%) to plasma proteins, mainly albumin and, to a lesser extent, *α*1-acid glycoprotein, and has a half-life of 11 to 17 h (Table 5). Micafungin is not a substrate for P-glycoprotein and does not affect its activity. It is metabolised by arylsulfatase, catechol-O-methyltransferase, and several cytochrome P450 (CYP) isoenzymes (3A4, 1A2, 2B6, and 2 C). It degrades to at least eleven metabolites that are excreted in the bile over many days. Human organism also disposes of them in the urine. Less than one percent of the drug is excreted unchanged via this route^94^^,^^95^. Micafungin is used to treat patients suffering from invasive candidiasis, candidemia, oesophageal candidiasis, abdominal abscesses, and peritonitis. It is used in the prophylaxis of *Candida* infections in patients undergoing haematopoietic stem cell transplantation or when a patient is expected to have granulocytopenia^96^. Studies show that it is possible to use micafungin as alternative antifungal prophylaxis in patients with acute leukaemia and myelodysplastic syndrome^97^. Micafungin also affects the immune system by potentiating its response by improving human macrophage activation^98^. A saturating dose is not required; doses of 100–150 mg daily provide minimum plasma concentrations of approximately 2–2.5 µg/ml on the first day of therapy^95^. Dosing in adults is 150 mg for oesophageal candidiasis, 100 mg for invasive candidiasis, and 50 mg for prevention of *Candida* infection (Table 6). In paediatric patients, the administered dose ranges from 1 to 4 mg/kg body weight depending on the disease present^53^.

### Anidulafungin

Anidulafungin is active against a wide range of *Candida* species, including those resistant to azoles, amphotericin B, and other echinocandins. It is less active against *C. guilliermondii* and *C. parapsilosis*^99^. Out of the echinocandins used, anidulafungin has the greatest activity against species of the genus *Aspergillus* even in the case of *Aspergillus lentulus*, which has reduced sensitivity to most antifungal drugs^100^. This antibiotic is highly bound to plasma proteins (99%) and has a half-life of 24–26 h in the human body (Table 5). Anidulafungin is non-enzymatically metabolised in human plasma during biotransformation processes to an open ring peptide lacking antifungal activity, which is excreted mainly in the faeces^99^^,^^101^. Since the degradation of the drug does not occur in the liver and the metabolites are mainly excreted biliary, the compound is safe for the treatment of individuals with liver and kidney failure^102^. As an antifungal antibiotic, anidulafungin is used to treat oesophageal candidiasis, candidemia, abdominal abscesses, and peritonitis in patients with or without neutropenia^103–105^. It is possible to treat pneumonia caused by *Pneumocystis jirovecii* with anidulafungin in patients who cannot tolerate trimethoprim or sulfamethoxazole^106^. Adult patients with invasive candidiasis receive a dose of 100 mg of anidulafungin per day after a 200 mg saturating dose, and for oesophageal candidiasis, 50 mg per day after a 100 mg saturating dose (Table 6). Infusion of the saturating dose should last approximately 3 h and the maintenance dose approximately 1.5 h^99^. Altered clearance and volume of distribution have been noted in patients weighing more than 140 kg which may result in less exposure to echinocandins in infected tissues. In that cases it is recommended to increase the antibiotic dose by 25% compared to the standard amount^95^. The plasma concentration of anidulafungin is maintained below 1 mg/l throughout the dosing period. Paediatric patients receive a saturating dose of 3 mg/kg body weight followed by a maintenance dose of 1.5 mg/kg body weight^5^^,^^21^. Liposome formulations of anidulafungin show greater efficacy compared to administration of the drug in free form. Such formulations could potentially (so far not approved by FDA) support treatment and reduce the occurrence of drug resistance^107^.

### Rezafungin

Rezafungin is the first representative of the second generation class of echinocandins^10^. It is effective against *Candida spp.*, *Aspergillus spp.*, *Trichophyton mentagrophytes*, *Trichophyton rubrum*, and *Microsporum gypseum*^50^. Rezafungin is also effective against isolates in which there is confirmed resistance to other echinocandins or azoles (*C. auris*, *C. parapsilosis*, *C. glabrata*)^48^^,^^73^. Rezafungin is currently in Phase III of clinical trials in patients with candidemia and invasive candidiasis^48^. The antibiotic is also effective in preventing *Pneumocystis spp.* pneumonia^23^. A study in a mouse model showed that 3 weeks of rezafungin prophylaxis was as effective as the standard treatment of pneumocystis pneumonia with trimethoprim or sulfamethoxazole^22^. Rezafungin also shows very potent activity against *A. fumigatus*, even against cryptic multidrug-resistant strains carrying a mutation in the *CYP51A* gene (causing azole resistance) which may suggest an effective treatment for azole-resistant aspergillosis^10^^,^^108^. Rezafungin binds strongly to proteins (99%), has a long half-life (80 h after the first dose, after subsequent dose 150 h), and its high safety profile allows for high doses (400 mg first dose, subsequent 200 mg) once a week^22^^,^^23^^,^^108^^,^^109^ (Table 5). The pharmacokinetics of rezafungin are relatively linear regardless of dose and its prolonged action enhanced drug penetration into infected tissues^22^^,^^110^. Rezafungin has a better safety profile compared to other echinocandins^10^. The solubility in water is above 150 mg/ml and in acetate, lactate and tris buffers is 45 mg/ml^15^. Rezafungin is rapidly distributed to the kidney and liver and its urinary excretion is negligible. It shows better distribution to hepatic abscess areas compared to micafungin (the concentration for rezafungin was 44.5 µg/g and for micafungin was 3.4 µg/g 24 h after drug administration)^111^. Rezafungin at a concentration of 0.25 µg/ml inhibits biofilm formation in *C. albicans* by deformation of the cells and release of their contents^10^^,^^112^.

Lyophilised rezafungin powder shows much less degradation during storage compared to other echinocandins. Storage of rezafungin at 40 °C, at room temperature in 5% dextrose, 0.9% NaCl solution or sterile water for more than 1 year showed minimal degradation of the compound (less than 7%) and no epimerization^10^^,^^15^^,^^23^. Infusion solutions can be stored without stabilisers and without fear of photolysis or spontaneous degradation^15^. Rezafungin can potentially be topical and subcutaneously applied^10^^,^^113^.

### Synergistic action of echinocandins with other antibiotics

Combinations of echinocandins along with other antifungal antibiotics are currently being investigated. Results of recent studies indicate an additive effect of caspofungin and voriconazole against an echinocandin-resistant strain of *C. glabrata*. Moreover, using the checkerboard test, combinations of caspofungin with azoles and amphotericin B showed an increase in fungicidal effect from 17.65% to 29.41% against this species^114^. The combination of anidulafungin and isavuconazole increases efficacy against azole-resistant *A. fumigatus*, which may benefit patients with invasive aspergillosis when azole therapy does not work^115^. Nikkomycin Z, along with caspofungin or micafungin, has shown enhanced activity against biofilms produced by *C. albicans* and *C. parapsilosis*. More effective activity against biofilms was shown by the combination of nikkomycin Z with micafungin compared to the combination with caspofungin^116^. The combination of colistin, which has no antifungal properties, with caspofungin resulted in increased potency against *C. auris*. This is presumed to be due to the alteration of the fungal cell wall structure by echinocandins, making it easier for colistin to interact with the fungal cell membrane^117^. Combination therapy with voriconazole and anidulafungin has also been shown to be equally effective and less costly in patients with a haemolytic disease or haematopoietic cell transplantation compared to voriconazole monotherapy^118^. New research suggests a synergistic effect of caspofungin and isavuconazole, but further studies are required^119^. Farnesol, which is a quorum-sensing molecule that enhances the effects of antifungal drugs, exhibits synergistic effects with echinocandins against *C. auris* biofilms^120^.

During studies on echinocandin-resistant species, compounds that potentiate their effects were also discovered. The most promising was DTPA (pentetic acid), which modulates echinocandin resistance phenotypes by chelating metal cations, especially magnesium and zinc excluding iron. This compound yielded beneficial relationships in mouse models of candidiasis. A chelator that potentiates the effects of echinocandins may represent a novel therapeutic strategy for combating drug-resistant fungi^121^.

## Side effects of echinocandins

Side effects of treatment with echinocandins are much milder compared to other antifungal antibiotics. Troublesome adverse reactions that force withdrawal occur less frequently with echinocandins than with other systemic antifungal drugs^18^. For example, amphotericin B administered intravenously can cause seizures, violent fevers, chills, myalgia, and hyperkalemia. In addition, the use of this antibiotic poses the likelihood of permanent kidney and liver damage due to its hepato- and nephrotoxicity^122^. The most common side effects associated with intravenous infusion of echinocandins are facial flushing, edoema, rash, pruritus, thrombophlebitis, bronchospasm, dyspnoea, decreased blood pressure, and fever^9^. The symptoms listed above can be seen with all echinocandins, but the incidence varies depending on the drug administered. Fever is a common side effect reported in approximately 35% of caspofungin-treated patients, whereas it is reported in only 1% of micafungin-treated patients. To reduce side effects, the rate of antibiotic infusion may be reduced^36^^,^^123^. Common disordered effects such as nausea, vomiting, and diarrhoea occur in 7% of patients, and 3–25% of patients treated with caspofungin are diagnosed with phlebitis. Less than 2% of patients experience these complaints after treatment with anidulafungin and micafungin^14^. Caspofungin shows a higher frequency of liver dysfunction (1–15%) compared to other echinocandins. Micafungin may increase risk of liver cancer^72^. Complications such as anaemia, neutropenia, leukopoenia, and thrombocytopenia comprise less than 10% of all adverse effects of echinocandins^9^. Echinocandins should be avoided during pregnancy because they exhibit embryotoxic and teratogenic effects^124^.

## Antiviral activity of echinocandins

Studies on micafungin have demonstrated antiviral activity against enterovirus 71 (EV71), chikungunya virus (CHIKV) and dengue virus serotype 2 (DENV-2)^125–127^. For the first time, micafungin as an antiviral agent was used against the proliferation and replication of enterovirus 71 (EV71) replicons with an IC50 (concentration of drug that reduce viral activity by 50%) value of 6.35 µg/ml^126^. Micafungin also has antiviral activity against other enteroviruses such as group B coxsackievirus type 3 (CVB3) and human rhinovirus (HRV). Studies on the effect of micafungin against CHIKV have provided information on the binding of the antibiotic to the virus envelope proteins. Micafungin may affect the later stages of viral infection. The drug reduces the cytopathic effect of the virus, its replication and impairs cell-to-cell transmission. The IC50 value of micafungin against CHIKV S27 ranged from 17.2–20.63 µM, which was higher than the IC50 value against EV71. Micafungin is also effective against other alphaviruses such as SINV and SFV^127^.

Recently, a pioneering study on the action of micafungin against dengue virus serotype 2 (DENV-2) has emerged. It is speculated that the mechanism of antiviral action of micafungin may be related to the degradation of the virion by binding the antibiotic to the envelope protein of DENV-2. The action of micafungin on the dengue virus results in the reduction of viral RNA levels. Micafungin mainly acts during the first stages of dengue virus infection. It inhibits DENV-2 binding and entry at doses ranging from 12.5–100 µM. Moreover, micafungin showed activity against other serotypes of the dengue virus (DENV-1, DENV-3 and DENV-4). Other echinocandins have also been shown to act on the dengue virus. Micafungin has the ability to suppress infections caused by arboviruses (CHIKV and DENV). The data presented by Chen et al. 2021 in the *in vitro* cellular model cannot be directly applied in clinical practice at this time therefore further studies require *in vivo* testing^125^.

## Resistance of fungi to echinocandins and “eagle-like effect”

Fungi possess adaptive mechanisms by which they compete with other microorganisms for the resources of the ecological niche they occupy. These adaptations can result in increased resistance to antifungal drugs^128^. Multidrug-resistant *Candida* species have spread worldwide, which in the future may affect methods used to treat infections^129^. Acquired resistance to echinocandins of various strains of *C. albicans*, *C. dubliniensis*, *C. kefyr*, *C. glabrata*, *C. krusei*, *C. tropicalis* and *C. lusitaniae* has been increasingly described. It is speculated that prolonged or repeated exposure to these antibiotics is a major factor in the acquisition of resistance by *Candida spp.* to echinocandins^130^.

**Figure 14.**
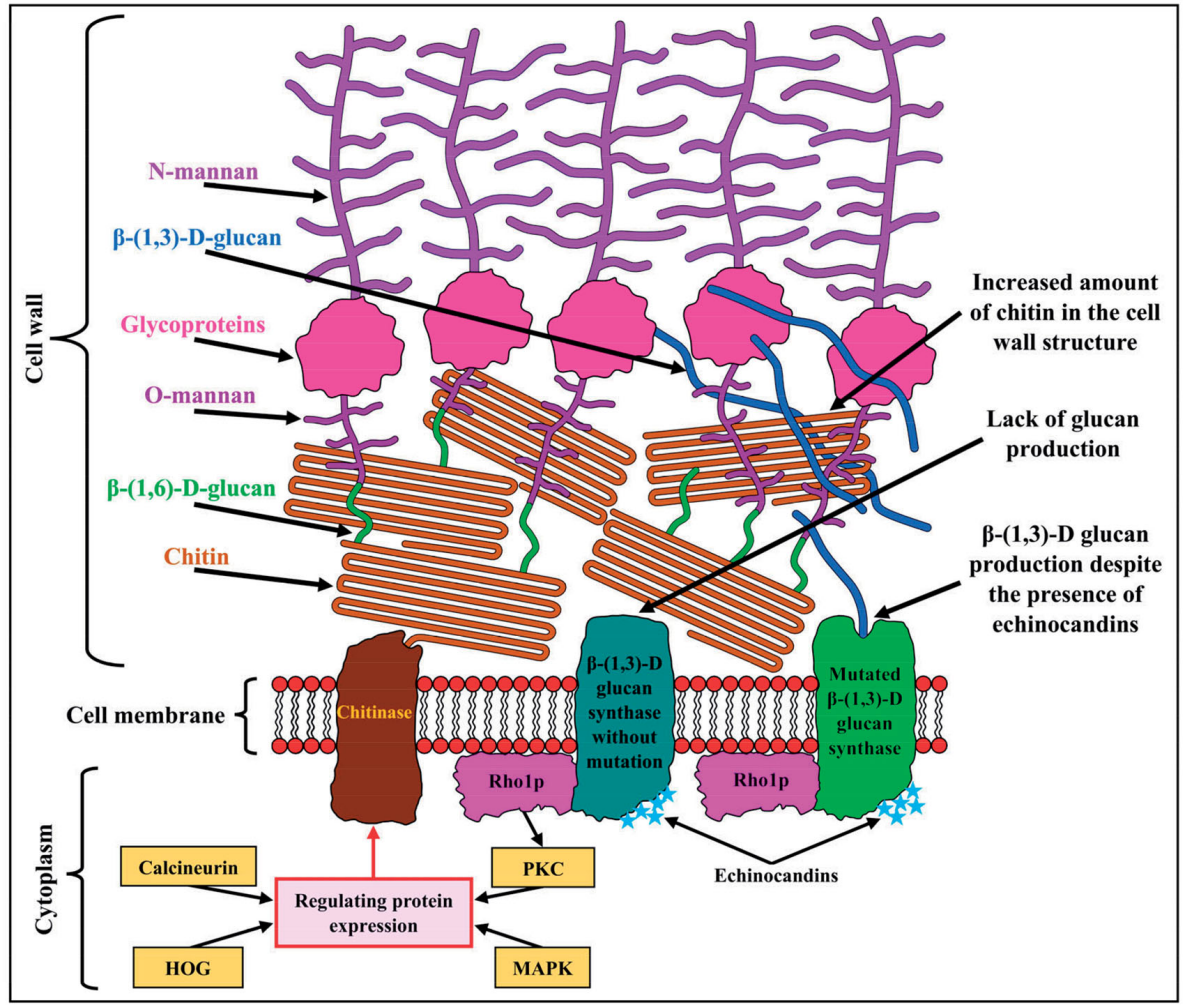
Mechanisms that adapt fungi to echinocandins. The protein kinase C (PKC), calcineurin, high-osmolarity glycerol (HOG) response, and mitogen-activated kinase (MAPK) pathways induce the synthesis of a compensatory cell wall composed of chitin. FKS mutations in the β-(1,3)-d-glucan synthase "hot spot" alter the enzyme's sensitivity to echinocandins and allow glucan production despite the presence of this antibiotic.

In response to echinocandins, fungi activate adaptive mechanisms that induce cell wall repair. Signals of cell wall instability are transmitted to the Rho1 subunit of β-(1,3)-d-glucan synthase, whose function is to control glucan synthase and coordinate the activity of protein kinase C (PKC)^131^. PKC controls the activity of other proteins responsible for maintaining the integrity of the fungal cell by synthesising a compensatory cell wall, which is mainly composed of chitin (Figure 14) ^132^. Increased levels of chitin synthesis in response to echinocandins can also be controlled by the mitogen-activated protein kinase (MAPK), high-osmolarity glycerol response (HOG), and calcineurin pathways^46^. Calcineurin, upon calcium activation, causes dephosphorylation of the protein transcription factor Crz1 (Calcineurin-Responsive Zinc Finger), which, upon translocation to the cell nucleus, induces *FKS2* expression by binding to calcium-dependent response elements (CDREs) in promoter sequences^46^^,^^131^.

Many studies on echinocandin resistance involve mutations in the catalytic subunit of glucan synthase (Fks). Three genes encoding this subunit are known: *FKS1*, *FKS2*, and *FKS3*. Variations in the gene encoding the Fks3 subunit affect the enzyme activity more weakly compared to the other genes in this group and therefore are not essential for the development of drug resistance^133^. Most *Candida* species acquire resistance to echinocandins through mutations in the Fks1 subunit gene, but in *C. glabrata*, resistance-causing mutations can also occur in the *FKS2* gene^134^. Acquired changes in the structure of the Fks subunit are mostly amino acid substitutions, but deletions and alternative "stop" codons are also found in *C. glabrata*^130^. Mutations that determine fungal resistance to echinocandins are located in two highly conserved regions of genes encoding the Fks subunit of glucan synthase called "hot spots" (HS). The point mutations are grouped into two "hot spot" regions: *HS1* encoding amino acids at positions 641–649 of the enzyme and *HS2* responsible for encoding amino acid residues 1345–1365 in the Fks1 protein in *Candida albicans* and homologous regions of Fks2 in *C. glabrata*. The *FKS2* "hot spot" mutation was also detected in echinocandin-resistant *S. cerevisiae* strains^133^. All members of the *C. parapsilosis* group (*C. parapsilosis*, *C. orthopsilosis*, and *C. metapsilosis*) have a natural DNA polymorphism at position Pro 649 of glucan synthase, resulting in a proline substitution by alanine, making them less susceptible to caspofungin. In *C. guilliermondii* these mutations are located at Met 663 and Ala 634 and serve similar results^135^. Glucan synthase gene sequence changes associated with the phenotype with the strongest resistance to echinocandins occur at positions encoding Ser 645 and Phe 641 and account for 80% of all mutations detected in *C. albicans* in the Fks1 subunit^136^. The most common and strongest phenotype substitution involves a change in serine at position 645 to phenylalanine, proline, or tyrosine^129^. The Ser 663 change in the Fks2 subunit in *C. glabrata* that is equivalent to Ser 645 in *C. albicans* is the most relevant amino acid substitution from the perspective of acquiring echinocandin resistance in this species^137^. Mutations in *A. fumigatus* occur in the *AfFKS1* gene of glucan synthase, which, along with increased chitin production, develops resistance of *Aspergillus* to echinocandins^138^.

Single amino acid substitutions in the glucan synthase sequence in various fungi reduce drug sensitivity by 50–3000-fold and increase MIC values by 5–100-fold. Amino acid substitutions in *C. albicans* Fks1 resulted in altered cell wall morphology^139^. *C. albicans* strains with a homozygous *FKS1* "hot spot" mutation exhibit thicker cell walls, which may be partially attributed to a compensatory increase in wall chitin content. Mutants with high chitin content in the cell wall show reduced growth rates in a liquid medium and impaired ability to transform blastospores into hyphae^140^. The increase in MIC values for echinocandins depends on the position and specific amino acid substitution in the glucan synthase structure. The most significant increase in MIC values was found for changes involving the first and fifth amino acids (phenylalanine and serine, respectively) in the "hot spot 1" regions of the genes encoding *FKS1* or *FKS2*. In most cases, *FKS* sequence changes cause cross-resistance to all echinocandins^130^. Resistance to echinocandins can change with the level of *FKS* gene expression in *C. albicans*^133^^,^^140^. *FKS2* expression in *C. glabrata* is calcineurin-dependent, meaning that echinocandin resistance can be reduced by including calcineurin inhibitors *FK506* in treatment^141^.

The observation of the specific strain of *C. albicans* which was able to grow in the presence of very high concentrations of caspofungin, significantly exceeding the MIC values, prompted attempts to explain this phenomenon^142^. It was found that a similar phenomenon can also be observed for other species such as *C. parapsilosis*, *C. glabrata*, *C. tropicalis* and *C. krusei*, as well as for other antibiotics from the echinocandins^143^. This paradoxical effect of fungal growth at very high concentrations of echinocandins is referred to as “Eagle-like effect" and is defined as the reduced activity of echinocandins against fungi when exposed to a dose well above the MIC. This effect is suspected to be due to adaptation as a result of stimulation of calcineurin pathways and chitin synthesis to maintain cell wall integrity^144^. Another hypothesis is the overproduction of polysaccharides that may complement β-(1,3)-D glucan deficiencies in the cell wall. "Eagle-like effect" is often observed in *Aspergillus* species (e.g. *A. fumigatus*) during exposure to high concentrations of caspofungin (above 1 mg/l)^143^. The described effect was found *in vitro* and during clinical caspofungin treatment of patients with invasive pulmonary aspergillosis^67^. The study showed that high doses of caspofungin on the order of 150 mg per day did not significantly improve treatment outcomes compared to patients treated with standard therapy (50 mg caspofungin per day). In addition, in cases of infections caused by *C. glabrata* and *C. tropicalis*, more failed treatments were reported at the 150 mg daily dose of caspofungin compared to the 50 mg daily dose, which may confirm the occurrence of the "Eagle-like effect" in clinical practice^145^.

## Conclusion

Echinocandins have become a good alternative to azoles and polyenes in the treatment of severe fungal infections caused in particular by *Candida* and *Aspergillus* species due to their unique mechanism of action and relatively mild side effects compared to other antifungal drugs. These antibiotics block the synthesis of one of the main components of the fungal cell wall (β-(1,3)-d-glucan) by binding to the Fks subunit of β-(1,3)-d-glucan synthase. Their action results in cell wall defects and the death of fungal cells. The metabolic pathway that echinocandins targets does not occur in human cells, which limits side effects. They are also administered to patients with a weakened immune system, e.g. people suffering from AIDS, cancer, neutropenia, as well as to transplant patients who are particularly at risk of fungal infections due to immunosuppressive therapy. On the other hand, echinocandins are administered only intravenously, which limits the treatment process only to hospital conditions. They are also embryotoxic so they cannot be administered to pregnant women. They are also not effective in the treatment of fungal infections caused by species with limited content of β-(1,3)-d-glucan in the cell wall.

However, taking into account the limited number of available antifungal agents and the characteristic chemical structure of echinocandins, the road opens up to various structural modifications, and thus to obtain new and effective drugs. An example is rezafungin, which is currently in Phase III clinical trials. Due to its improved pharmacokinetics as well as better stability in solutions, it is a good example of the development of this class of antibiotics. Despite the relatively short history of medical use, echinocandins have been approved by the FDA and EMA as first-line drugs for oesophageal candidiasis, invasive candidemia and the prevention of mycoses in transplant patients, which undoubtedly confirms their high usefulness in the fight against mycoses. The cost of one dose of the drug varies between ± $50 (caspofungin) and ± $100 (anidulafungin, micafungin), which makes the therapy relatively expensive, but the increasing use of these drugs as well as the introduction of new preparations from this class may reduce the cost of therapy in the near future. Fungal resistance to echinocandins is found relatively rarely compared to other antifungal antibiotics, yet we already know the basic mechanisms of this phenomenon, which may help in the work on the synthesis of new representatives of more effective echinocandins.

Summarising this basic review of information on echinocandins, it can be stated that despite the relatively short period of their use, these drugs have already found a significant place in clinical practice and are now a very promising and developing class of antifungal antibiotics.
